# Why do hypertensive patients of African ancestry respond better to calcium blockers and diuretics than to ACE inhibitors and β-adrenergic blockers? A systematic review

**DOI:** 10.1186/1741-7015-11-141

**Published:** 2013-05-30

**Authors:** Lizzy M Brewster, Yackoob K Seedat

**Affiliations:** 1 Departments of Internal and Vascular Medicine, F4-222, Academic Medical Center, Meibergdreef 9, Amsterdam, AZ, 1105, The Netherlands; 2 Nelson R Mandela School of Medicine, Faculty of Health Sciences, University of KwaZulu Natal, Private Bag. 7, Congella, Durban, 4013, South Africa

**Keywords:** African ancestry, Antihypertensive therapy, Systematic review, Nitric oxide, Creatine kinase

## Abstract

**Background:**

Clinicians are encouraged to take an individualized approach when treating hypertension in patients of African ancestry, but little is known about why the individual patient may respond well to calcium blockers and diuretics, but generally has an attenuated response to drugs inhibiting the renin-angiotensin system and to β-adrenergic blockers. Therefore, we systematically reviewed the factors associated with the differential drug response of patients of African ancestry to antihypertensive drug therapy.

**Methods:**

Using the methodology of the systematic reviews narrative synthesis approach, we sought for published or unpublished studies that could explain the differential clinical efficacy of antihypertensive drugs in patients of African ancestry. PUBMED, EMBASE, LILACS, African Index Medicus and the Food and Drug Administration and European Medicines Agency databases were searched without language restriction from their inception through June 2012.

**Results:**

We retrieved 3,763 papers, and included 72 reports that mainly considered the 4 major classes of antihypertensive drugs, calcium blockers, diuretics, drugs that interfere with the renin-angiotensin system and β-adrenergic blockers. Pharmacokinetics, plasma renin and genetic polymorphisms did not well predict the response of patients of African ancestry to antihypertensive drugs. An emerging view that low nitric oxide and high creatine kinase may explain individual responses to antihypertensive drugs unites previous observations, but currently clinical data are very limited.

**Conclusion:**

Available data are inconclusive regarding why patients of African ancestry display the typical response to antihypertensive drugs. In lieu of biochemical or pharmacogenomic parameters, self-defined African ancestry seems the best available predictor of individual responses to antihypertensive drugs.

## Background

There is a great need for individual treatment options in hypertensive patients of African ethno-geographical ancestry [1-5]. Compared with hypertension in other population subgroups, the disorder in these patients is often more severe, more resistant to treatment, and leads to earlier end organ damage and premature death [1-4]. Thus, hypertension seems to be a more aggressive disease in patients of African ancestry. This has important implications for the choice of an antihypertensive agent [3,5].

Antihypertensive drugs were the first cardiovascular therapy for which there was wide recognition of differences in clinical efficacy related to ethno-geographical ancestry [6]. Patients of African ancestry as a group respond better to calcium blockers and diuretics, while the response to β-adrenergic blockade and inhibition of the angiotensin converting enzyme is attenuated (Table 1) [3,5,7]. However, there is considerable interindividual variation in this response [7,8].

**Table 1:** Differences in clinical efficacy of antihypertensive drugs in ancestry groups

	Systolic/diastolic blood pressure reduction*		
**Drug category**	**European ancestry**	**African ancestry**	**Difference**^**†**^
**Calcium blockers**	15.3/12.6	16.9/13.3	2.4/0.6
	(14.7, 15.9)/(12.3, 12.9)	(16.0, 17.7)/(12.9, 13.8)	(3.4, 1.3)/(1.2, 0.0)
**Diuretics**	11.5/9.1	15.0/10.7	3.5/1.5
	(9.5, 13.4)/(8.1, 10.1)	(13.1, 17.0)/(9.5, 11.9)	(6.4, 0.5)/(3.1, −0.1)
**ACE-i**	12.8/11.4	8.5/8.0	−4.6/−3.0
	(11.7, 13.9)/(10.8, 12.0)	(7.0, 9.9)/(7.1, 8.9)	(−2.7, −6.5)/(−1.9, −4.1)
**β-Blockers**	11.7/11.3	5.9/9.5	−6.0/−2.9
	(10.2, 13.3)/(10.5, 12.1)	(4.2, 7.6)/(8.5, 10.4)	(−3.6, −8.3)/(−1.6,−4.2)

Legend: Data depicted are pooled estimates (95% confidence intervals) from systematic reviews [3,7]. ACE-i, angiotensin converting enzyme inhibitors. *Mean blood pressure reduction (mm Hg). ^†^The depicted difference is the weighted pooled difference in response between ancestry groups, with positive values indicating a greater response in patients of African ancestry and negative values indicating a greater response in patients of European ancestry.

Greater knowledge about the potential causes for these differences might lead to more individualized treatment regimens, but to our knowledge, no previous study has systematically addressed why patients of African ancestry may have this specific pattern of responses. The aim of this paper is to provide a systematic overview of the factors associated with the differential drug response of patients of African ancestry to antihypertensive drug therapy.

## Methods

We sought to identify all published or unpublished studies that considered potential explanations for the differential clinical efficacy of different classes of antihypertensive drugs, used as single drug or single drug-based treatment in non-pregnant adults of sub-Saharan African descent with uncomplicated hypertension, defined as the absence of reported clinical heart failure, current stroke or end stage renal disease.

We first identified potential causes for differences in specific drug responses based on ethno-geographic origin (Table 2). As we sought to explain differential blood pressure lowering responses to different types of antihypertensive drugs, we excluded general factors such as access to care and differences in socio-economic status. To answer the clinical question, why there was a difference in response between people of African vs European ancestry, we considered pharmacokinetic variations including polymorphisms in cytochrome P450 family of enzymes involved in phase I drug metabolism, and polymorphisms in genes encoding enzymes involved in phase II drug metabolism. Furthermore, we considered genetic polymorphisms that may influence pharmacodynamics including alpha-adducin (*ADD1*), subunits of G-proteins (*GNB3* and *GNAS1*), the β-1-adrenergic receptor (*ADRB1*), endothelial nitric oxide synthase (*NOS3*), and components of the renin-angiotensin-aldosterone (RAAS) system, angiotensinogen (*AGT*), renin (*REN*), angiotensin converting enzyme (*ACE*), the angiotensin II receptor type I (*AGTR1* or *AT1R*), and aldosterone synthase (*CYP11B2*) [9]. Finally, hypertension in persons of African ancestry is characterized by high vascular contractility, greater salt sensitivity and, in general, low plasma renin activity [2], and the molecular basis of these changes has been related to low nitric oxide (NO) bioavailability [10], to the activity of Ca^2+^ATPase, myosin ATPase, Na^+^K^+^ ATPase, and to the central regulatory enzyme of energy metabolism, creatine kinase (CK), which rapidly regenerates adenosine triphosphate (ATP) from phosphocreatine near these ATPases [11,12].

**Table 2:** Factors that may affect the differential drug response of patients of African ancestry

**Category**	**Factors**
Environmental	Diet (sodium) [2]
Bioavailability	Absorption, First pass metabolism (intestinal and phase 1 drug metabolism, polymorphisms cytochrome P450 enzymes, phase 2 drug metabolism) [6,9]
Distribution	Protein binding, distribution volume
Receptor	Receptor sensitivity and genetic variation [6,9]
Hemodynamics	Low renin, sodium-volume dependent hypertension [2]
Intracellular effects	Nitric oxide, cGMP, cAMP, calcium fluxes, ion transport, rho kinase, creatine kinase, myosin light chain kinase, myosin ATPase [10-12]
Elimination	Kidney, liver or other route

Using these environmental, pharmacokinetic, pharmacodynamic and pharmacogenomic factors, we conducted a systematic literature search in electronic databases, including PUBMED, EMBASE, LILACS (Literatura Latino-Americana y del Caribe en Ciencias de la Salud), the African Index Medicus (AIM), and the Food and Drug Administration (FDA) and European Medicines Agency (EMA) databases, dated June 2012.

We developed a search strategy to find papers that considered causes for differential responses, rather than finding clinical trials *per se*[3]. To reach this end, the most effective strategy in terms of the yield in eligible papers was to not include drug names, or “hypertension”, but the factors as mentioned in Table 2, using the following keywords: “(salt OR pharmacokinetic OR resorption OR bioavailability OR liver OR first pass OR metabolism OR cytochrome OR n-acetyltransferase OR catechol-o-methyltransferase OR phenol sulfotransferase OR distribution OR protein binding OR elimination OR pharmacodynamic OR pharmacogenetic OR receptor OR G-protein OR alpha-adducin OR nitric oxide OR c-GMP OR cAMP OR sarcoendoplasmic OR calcium OR ion OR creatine kinase OR rho kinase OR “myosine ATPase” OR “myosin light chain kinase”) and (black* OR Afr* OR Creole OR Carribean OR Caribbean OR negr* OR ethnic*) and antihypertensive.”

Finally, we hand-searched for studies by using electronic cross referencing (“related citations”) from PUBMED, references from textbooks, narrative reviews and systematic reviews; by contacting experts; and by searching the Internet. We did not restrict the searches to any specific language.

To produce a rigorously conducted narrative systematic review, we used the “narrative synthesis approach” (the PRISMA guideline is not designed for narrative systematic reviews) [13]. This recently developed methodology is applied when one expects considerable heterogeneity among the studies of interest. Distinctively, a narrative rather than a statistical summary of the findings of studies is used to perform the data synthesis, which yields a more detailed analysis of heterogeneous data with less loss of information [13].

Any experimental research that is reported in the manuscript has been performed with the approval of an appropriate ethics committee. Research carried out on humans were in compliance with the Helsinki Declaration, and experimental research on animals followed internationally recognized guidelines.

## Results

### Paper flow

**Figure 1:**
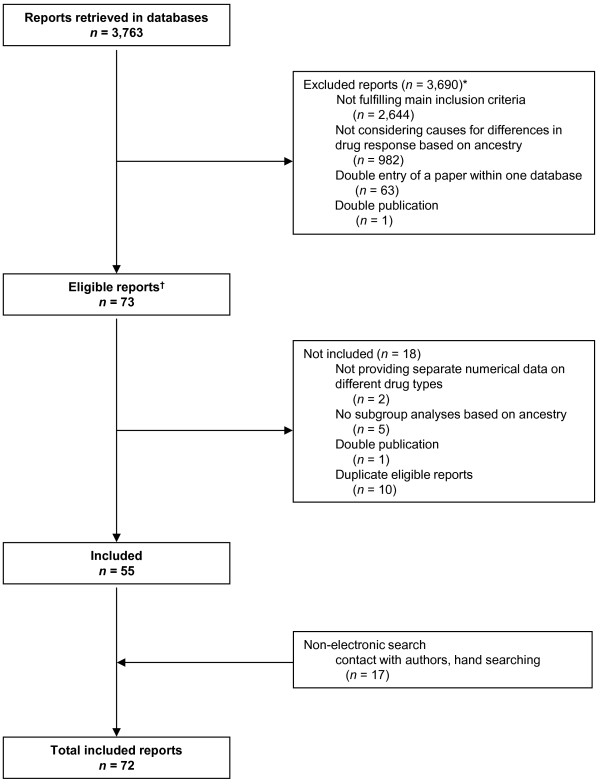
**Flow diagram.** Data were retrieved from PUBMED, EMBASE, LILACS, the African Index Medicus, and the Food and Drug Administration and European Medicines Agency databases. *Studies were excluded using a hierarchical approach. First, we excluded reports that did not fulfill the main inclusion criteria (n = 2,644): an original report considering drug therapy with different available drug types in non-pregnant adults of African ancestry with uncomplicated hypertension, defined as the absence of clinical heart failure, stroke or end stage renal disease as reported by the authors. Studies conducted exclusively in diabetics were also excluded in this step. Of the remaining studies fulfilling these main inclusion criteria (n = 1,119), most studies were excluded in the next step (n = 982), because these were not original reports providing an explanation for the difference in response to antihypertensive drugs between ancestry groups. As a quality and consistency check, each paper retrieved from the search yield (n = 3,763) was categorized, per database, thus the excluded paper categories harbor duplicate reports, occurring in more than one database. ^†^ Eligible reports thus fulfilled the inclusion criteria, and were original reports considering potential causes for the differential response of patients of African ancestry to antihypertensive drugs used as single drug or single drug-based treatment. Included studies from the electronic searches (n = 55) [14-68], and hand search (n = 17) [12,69-84] are described in detail in the Results section.

We retrieved 2,520 citations in PUBMED, 1,002 in EMBASE, 4 in LILACS, 2 in the AIM, 2 in the FDA and 229 in the EMA database for a total of 3,759 citations. Four citations in the EMA database contained 2 clustered reports, adding 4 papers to yield a total of 3,763 papers. After removing duplicate reports and applying the inclusion criteria, 55 papers were included from the electronic searches (please see Paper Flow, Figure 1, with detailed mention of the reason for exclusion) [14-68]. EMBASE, LILACS, AIM and the FDA database did not yield any additional included reports beyond the papers included from PUBMED, but one additional paper was included from the EMA database [68]. The majority of the excluded reports did not provide an explanation for differences in antihypertensive drug response related to ancestry. Hand search yielded 17 more papers [12,69-84], most of which had no ancestry/ethnicity/race tag in PUBMED, or were not indexed, such that these could not be retrieved with electronic searches. We did not use language restriction, but all included papers were written in the English language.

### Description of included studies

The included studies were original reports that provided, or attempted to provide, an explanation for the differences in antihypertensive drug responses between hypertensive patients of African and European ancestry. The design of the included studies varied, from observational studies to small and large scale clinical trials, in subjects of sub-Saharan African descent, studied within Africa (Nigeria [46,59,67], Kenya [64], and South Africa [26,61,62,69,78]), or in the African diaspora (the Netherlands, persons from Suriname, the Dutch Antilles, and Ghana [12,72,73]; United Kingdom, persons from Nigeria [54,60], Sierra Leone [54], Zimbabwe [54], Zambia [54], Tanzania [54], or country of origin not stated [50]; all other were in the United States, except for one paper that did not state the location of the study [34]). Ethno-geographic origin was either self-defined, or defined by the authors of the reports, in the participants being of European or African ancestry. Authors used different nomenclature for African descent, including black people, blacks, black race, black skinned people, African-Americans and Afro-Caribbeans; as well as for European descent, including white and Caucasian. We unified this to: ‘persons (or patients) of African ancestry’ versus ‘European ancestry’, throughout this paper, as this nomenclature captures concepts of genomic variation, biology or geographic history [85]. The majority of papers retrieved considered the four major classes of antihypertensive drugs: calcium blockers, diuretics, drugs that interfere with the renin-angiotensin-aldosterone system, and β-adrenergic blockers (Table 3). Data are synthesized below [13].

**Table 3:** Summary of findings

**Drug**	**High sodium diet**	**Pharmacokinetics**	**Pharmacodynamics**
**Ca-blockers**	No effect* on BP lowering efficacy [34,40,41,43]	1) Lower clearance nifedipine with African ancestry [46]	1) Ancestry/age profiling superior to renin in predicting drug response [38]
		2) *CYP3A4* genotypes sooner at BP goal^†‡^	2) Ca-blockers effectively block enhanced Ca-dependent vascular contractility, potentially mediated by high CK/low NO with African ancestry (Figure 2) [11,12,72]
		3) *CYP3A5* genotypes not associated with BP response [17,24]	3) Pharmacogenomics: *ACE* G12269A, C17888T, and G20037A, and variants in the promoter region of the angiotensinogen gene (−217G = > A and –20A = > C), were not associated with BP response to respectively amlodipine and nifidipine [23,26]
**Diuretics**	No effect on BP lowering efficacy [70]	No differences found between ancestry groups [33]	1) No association with plasma renin levels [57,63,65,66], or ancestry/age better predictor of response than renin [15,38]
			2) Diuretics effectively block enhanced sodium retention [86], potentially mediated by high CK in persons of African ancestry (Figure 3) [11,12,73,87]
			3) Pharmacogenomics: greater BP response with *AGT* 6A and *AT1R* 1166A alleles (only women); [30]*GNB3*T allele associated with greater BP response to HCT (only men); [32]*ACE* I/D, *CYP11B2* C-344 T, *REN* A7174G [30], *STK39*[76], α-adducin Gly460Trp, ADRBK1, and *GRK5* Gln41Leu [77] not associated with BP response
**ACE-i**	Lower efficacy with high salt [41]	No association of BP response with *CYP3A4* A392G, T16090C, or *CYP3A5* A6986G genotypes [17]	1) Ancestry/age profiling superior to renin in predicting drug response [38]
			2) Low NO bioavailability may attenuate response (Figure 2) [10,12,36,37,72,79-81]
			3) Pharmacogenomics: *ACE* DD poorer response to lisinopril;[28]^§^ Homozygous *ACE* G12269A and C17888T faster on BP goal with ramipril than heterozygous genotypes; [23] AA genotype 217G = > A and –20A= > C, promoter region of the angiotensinogen gene: no significant BP decrease with enalapril or lisinopril [26].
**β-Blockers**	No effect on BP lowering efficacy [70]	No consistent differences between persons of African vs European ancestry [44,45,52,55,56,59,61,64],[67]	1) Ancestry/age profiling superior to renin in predicting drug response [15,38,53]
			2) High vascular contractility may promote peripheral vasoconstriction with β-adrenergic blockers (Figure 2) [3,11,12,72,88-92]
			3) Pharmacogenomics: *ADRB1* Arg 389/Ser 49 associates with greater, or attenuated BP lowering; [14,20,74]*GRK4* Ala142Val faster on BP goal with metoprolol (only men); [19]*GRK4* Arg65Leu and Ala486Val, *GRK5* and *GRK2* genotypes not associated with BP response [18,77]

Legend: Diuretics, hydrochlorothiazide (HCT), or other diuretic drug; ACE-i, ACE inhibitors; β-blockers, β-adrenergi c blockers; BP, blood pressure; Ca-blockers, calcium channel blockers; CK, creatine kinase. *At higher drug dose; ^†^Pharmacodynamics unclear; ^‡^Only women/usual BP goal with *CYP3A4* A392; or low BP goal with *CYP3A4* 16090C. ^§^Very modest effect, −0.85 mm Hg systolic (SE 0.51) and −0.50 mm Hg diastolic (SE 0.28).

### Narrative synthesis

#### Calcium blockers

##### Clinical efficacy

Calcium blockers are with diuretics among the most effective classes of drugs to reduce blood pressure in patients of African ancestry [3,7]. This drug type remains effective in all subgroups of sex, age and blood pressure strata, including high baseline diastolic blood pressure (>/= 110 mm Hg). Side effects include headache and ankle edema [3,7].

##### Environmental factors

Calcium antagonists manifest a more robust blood pressure lowering effect, even in the setting of salt intake *ad libitum* or a high sodium intake, albeit at the expense of a higher drug dose [34,40,41,43]. When controlled, sodium intake in the studies varied between 40 to 100 mmol/day in low salt, and 190 to 300 mmol/day in high salt conditions [34,41,43]. With a high salt diet and isradipine, mean systolic blood pressure (SD) in hypertensive patients of African ancestry (n = 42) was: placebo 155.2 (19.3) vs. isradipine 139.3 (15.0) mm Hg; a difference of −15.9; and in patients of European ancestry (n = 92) placebo 156.9 (14.5) vs isradipine 142.1 (13.0); a difference of −14.8. With low salt, systolic blood pressure in patients of African ancestry was placebo 142.9 (17.0) vs isradipine 135.8 (15.6); a difference of −7.1; and in patients of European ancestry placebo 143.5 (14.6) vs isradipine 135.9 (12.3), a difference of −7.6 [40]. In addition, with high salt intake, the mean blood pressure lowering effect of calcium blockers exceeded the effect of ACE inhibitors in patients of African, but not of European ancestry [41].

##### Pharmacokinetics

Nifedipine clearance is reported to be lower in persons of African ancestry, with a 150% greater area under the plasma concentration-time curve; and a 79% higher elimination half-life [46], but no significant differences were found for nitrendipine [58].

Regarding genetic polymorphisms and pharmacokinetics, verapamil is a cytochrome CYP3A substrate, and CYP3A5 is thought to convert cortisol to 6 b-hydroxycortisol in the kidney, and to be associated with salt-sensitive hypertension. In the *CYP3A5* gene, the A4G (*3) and G4A (*6) polymorphisms result in severely decreased expression of CYP3A5 enzyme relative to a normal functional allele (*1) [24]. These polymorphisms were studied in the International Verapamil/trandolapril Study (INVEST) Genetic Substudy (INVEST-GENES), which included hypertensive subjects with coronary artery disease (n = 537; 43 of African ancestry). However, no association was found with the antihypertensive response to verapamil [24]. Amlodipine is also extensively metabolized in the liver, mainly by CYP3A4 and possibly CYP3A5. In the African-American Study of Kidney Disease and Hypertension (AASK), 1,094 self-identified African-American men and women between 18 and 70 years, diagnosed with hypertensive kidney disease (glomerular filtration rate between 20 and 65 ml/min per 1.73 m^2^), were randomized to amlodipine, ramipril or metoprolol, and a mean goal arterial blood pressure (MAP) of either 102 to 107 mm Hg (usual MAP goal) or ≤92 mm Hg (low MAP goal) to assess the effect on the decline in kidney function. Of these, 159 participants were analyzed for *CYP3A4* and *CYP3A5* polymorphisms. Only women randomized to a usual MAP goal, and with an A allele at *CYP3A4* A392G, were more likely to reach a target MAP of 107 mm Hg (adjusted hazard ratio of AA/AG compared to GG: 3.41 (95% CI: 1.20 to 9.64; *P* = 0.02). Among participants randomized to a lower MAP goal, men and women with the C allele at *CYP3A4* T16090C were more likely to reach the target MAP of 107 mm Hg (adjusted hazard ratio 2.04 (95% CI 1.17 to 3.56; *P* = 0.01). C*YP3A5* A6986G was not associated with blood pressure response in this study [17].

##### Pharmacodynamics

Profiling using age and ancestry was shown to be superior to renin levels in predicting the magnitude of the antihypertensive response to diltiazem [38]. Calcium blockers’ main effect is vasodilation through a direct effect on the smooth muscle layer of resistance arteries [31]. The drugs reach their effect through a reduction of the intracellular calcium concentration in smooth muscle, by the blocking L-subtype, voltage-sensitive, slow calcium channels in cell membranes and calcium outflow from the sarcoendoplasmic reticulum [3,11,93].

**Figure 2:**
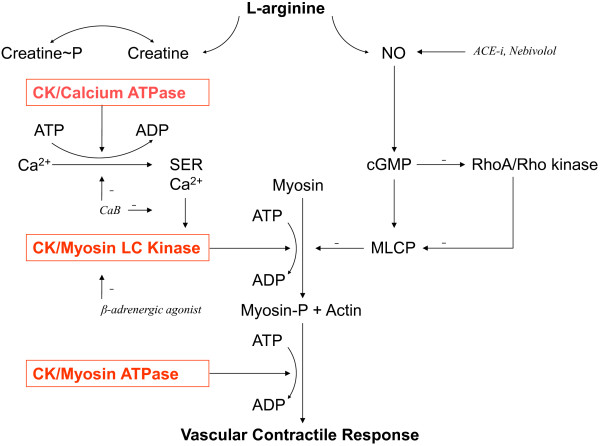
**Modulators of vascular contractility.** This is a schematic representation of the main regulatory pathways of vascular smooth muscle contraction, based on Brewster *et al.*[12,72]. Creatine kinase (CK) is colocalized with Ca^2+^ ATPase and myosin ATPase, and evidence suggests the enzyme is also colocalized with myosin light chain (LC) kinase, to rapidly supply these enzymes with ATP using creatine phosphate (Creatine ~ P) [11,12,72,88-90]. The guanidino compounds creatine and nitric oxide (NO) have a common precursor in L-Arginine [12]. NO, RhoA/Rho kinase, and calcium-dependent pathways are intracellular effectors of blood pressure-regulating systems that converge on metabolic processes fueled by CK [11,12,72,88-91,93-95]. CK is high in persons of African ancestry [11,12,72,73], and this is thought to lead to greater contractility of vascular smooth muscle [11,12,72]. Vascular contractile responses can be reduced through enhancing NO-dependent pathways, including with ACE inhibitor (ACE-i) or nebivolol-induced NO synthesis, or through indirect inhibition of CK-dependent pathways, as with calcium blockers (CaB) or β-adrenergic agonists. Calcium blockers may block the entry of calcium in the cell as well as the outflow from the sarcoendoplasmic reticulum (SER) [93]. β-adrenergic agonists reduce contractility mainly through inhibition of myosin light chain kinase [91]. β-adrenergic blockers antagonize this beneficial effect, which may help explain the more frequent occurrence of blood pressure increase with β-blockers in persons of African ancestry [3,92], within the context of the greater vascular contractility in this population subgroup [11,12,36,37,39,72]. cGMP, guanosine cyclic 3′,5′-(monophosphate); MLCP, myosin light chain phosphatase.

The high efficacy of calcium blockers in patients of African ancestry points to enhanced vascular smooth muscle contractility in this group [11,12,36,37,39,72]. This is thought to be a result of a “double jeopardy”: a lack of NO bioavailability [10,12,31,36,37,72,80], and related high activity of the enzyme CK [10,12,72] (Figure 2). CK fuels Ca^2+^ ATPase at the sarcoendoplasmic reticulum and, thereby, calcium uptake, as well the ATPases directly leading to vasoconstriction [12,72]. Thus, high vascular CK increases vascular contractility, as a final cellular step [72]. Furthermore, the high creatine demand associated with high creatine kinase might induce a relative lack of L-arginine and NO [12].

Therefore, the clinical efficacy of calcium blockers in patients of African ancestry may depend on the strong antagonistic effect of the drug on the enhanced vascular contractility induced by high CK and low NO (Figure 2), but there are no clinical data yet showing this to predict the response to calcium blockers.

Pharmacogenomic factors were studied in the AASK study, where G12269A, C17888T and G20037A *ACE* polymorphisms were not associated with blood pressure lowering in participants randomized to amlodipine (n = 159) [23]. In addition, in a study including patients of African ancestry (n = 108), functional variants in the promoter region of the angiotensinogen gene (−217G = > A and −20A = > C), which influence the transcription of the gene, did not predict the response to nifedipine [26].

#### Diuretics

##### Clinical efficacy

Diuretics are among the most effective blood pressure lowering drugs in patients of African ancestry [3,7], although there is evidence that with high baseline diastolic blood pressures, calcium blockers are more effective [3]. Furthermore, there are concerns regarding metabolic side effects, including abnormal glucose tolerance. This might be of particular importance to patients of African ancestry, who have a higher risk of developing diabetes, and often need to start treatment at a younger age [3].

##### Environmental factors

In the Trial of Antihypertensive Interventions and Management (TAIM), 692 participants (224 of African ancestry) aged 21 to 65 years, with diastolic blood pressure between 90 and 100 mm Hg and weight between 110% and 160% of ideal weight were randomized into diet (usual, low sodium-high potassium, weight loss) and drug (placebo, 25 mg/day chlorthalidone or 50 mg/day atenolol) groups resulting in nine diet plus drug combinations. When comparing subjects randomized to chlorthalidone (n = 24) vs placebo (n = 26) for usual vs low-sodium diet, adding sodium restriction (mean 100 mmol/day) to the diuretic drug did not enhance the blood pressure lowering effect [70].

##### Pharmacokinetics

No differences were found in bioavailability or elimination of hydrochlorothiazide between ancestry groups [33].

#### Pharmacodynamics

The activity of the renin-angiotensin-aldosterone system is thought to be inversely related to the blood pressure response to diuretics [30]. Therefore, renin profiling was used to predict the response to hydrochlorothiazide in six papers, 25 mg/d in 363 participants (152 of African ancestry), of the Pharmacogenomic Evaluation of Antihypertensive Responses (PEAR) study; [15] 12.5 to 50 mg/d in a Veterans Administration study in 335 subjects (152 of African ancestry); [38] 50 mg/d in 83 patients of African ancestry; [57] 50 to 200 mg/d in 212 participants (129 of African ancestry) in another Veterans Administration study; [63] 100 mg/d in 61 patients of African ancestry; [65] and 100 mg/d (vs furosemide 80 mg/d) in 29 patients of African ancestry [66].

Renin did not predict the response to hydrochlorothiazide monotherapy in four studies [57,63,65,66], nor to furosemide (80 mg/d) [66], or spironolactone 100 to 400 mg/d [65]. In the PEAR study, the β ± SE for prediction of systolic blood pressure with renin was 1.87 ± 0.90 (*P* = 0.04), with a relative contribution of African ancestry of −2.12 ± 1.47 (*P* = 0.15); [15] and regression models that included ancestry and age explained similar [15] or greater [38] variation in blood pressure response than renin.

**Figure 3:**
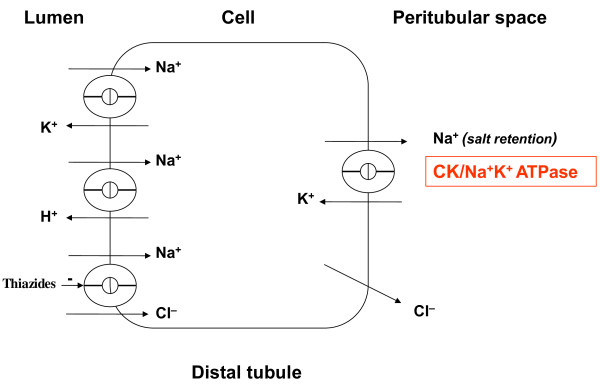
**Pharmacodynamics of thiazide diuretics.** This is a schematic reproduction of the kidney distal convoluted tubule. Sodium retention is driven by basolateral Na^+^K^+^ ATPase throughout the kidney [86]. Creatine kinase (CK), reported to be high in persons of African ancestry [11,12,72,73], is tightly bound near basolateral Na^+^K^+^ ATPase, where it rapidly regenerates ATP to facilitate sodium retention [87]. Enhanced sodium retention occurs more frequently in persons of African ancestry [96]. Thiazide diuretics counteract this effect, albeit indirectly and partly, through inhibition of luminal Na^+^Cl^−^ -cotransport.

Persons of African ancestry are reported to have a greater tendency to retain salt [2,11,96]. This is thought to be a primary renal mechanism, as the increased Na^+^ retention does not appear to be secondary to increased production of aldosterone, deoxycorticosterone, cortisol or 18-hydroxycortisol [82]. The main mode of action of thiazide diuretics is to inhibit Na^+^Cl^−^-cotransporter activity in the renal distal convoluted tubule, blocking sodium reabsorption across the luminal membrane. All sodium absorption throughout the kidney is energetically and osmotically driven by the basolateral sodium pump Na^+^K^+^ ATPase [86]. ATP generation to this sodium pump is supported by CK, which is tightly bound near Na^+^K^+^ ATPase to rapidly regenerate ATP *in situ*[87]. CK is thus thought to directly provide ATP for sodium reabsorption [11,87] (Figure 3).

The high CK activity in persons of African ancestry has been linked to the greater tendency in this group to retain salt [11,96]. Also, evidence was found for a more active Na^+^K^+^_2_Cl^−^-cotransporter in the thick ascending limb in persons of African ancestry [71]. This might render drugs that counteract sodium retention as a mode of action to be highly effective, but further data are needed to substantiate how differences in kidney function in persons of African vs European ancestry may impact responses to antihypertensive drugs.

In a pharmocogenomic approach, the association between variation in five structural genes encoding components of RAAS and the effect of monotherapy with hydrochlorothiazide 25 mg daily for four weeks, was studied in 255 hypertensive men and women of African and 246 European ancestry, including *AGT* (angiotensinogen) G-6A, *AT1R* (angiotensin II receptor, type 1) A1166C, *ACE* Insertion/Deletion; *CYP11B2* (aldosterone synthase) C-344 T, and *REN* (renin) A7174G. Only in women of African ancestry, but not in men or patients of European ancestry, blood pressure lowering was greater with an increasing number of AGT–6A alleles (−11.3 mm Hg for GG, −18.2 mm Hg for AG and −22.2 mm Hg for AA; *P* = 0.03); and of *AT1R* 1166A alleles (−14 mm Hg for CC, −15.6 mm Hg for AC and −22.5 mm Hg for AA; *P* = 0.04) [30].

The association between the response to hydrochlorothiazide 25 mg daily for four weeks and a polymorphism (C825T) in exon 10 of the gene encoding the b3-subunit of G proteins (*GNB3*), which potentially leads to enhanced sodium-proton antiport activity was assessed in men and women of African (n = 197) and European ancestry (n = 190) [32]. Relative frequencies of the CC, TC and TT genotypes of *GNB3* differed significantly between groups, with the T allele significantly more frequent in patients of African than in European ancestry (76.1% versus 28.9%, *P* <0.01). In patients of European ancestry, and in men, but not in women of African ancestry, the presence of the T allele was associated with a greater reduction in systolic and diastolic blood pressure with treatment, with mean declines of respectively, 10.2 and 5.9 mm Hg in CC; 13.6 and 7.8 mm Hg in TC; and 16.3 and 10.5 mm Hg in TT genotypes, accounting for 3.1% and 4.5%, respectively, of interindividual variation in the systolic and diastolic blood pressure responses to hydrochlorothiazide [32].

The WNK-SPAK-Na^+^Cl^−^-cotransporter pathway has been previously implicated in thiazide response, as variations in *WNK1* were associated with differential BP response to hydrochlorothiazide [76]. Therefore, 195 “good” and 194 “poor” responders to hydrochlorothiazide 25 mg daily from the Genetic Epidemiology of Responses to Antihypertensives study were genotyped for approximately 100 single nucleotide polymorphisms within 5,000 bases of *STK39*, with a replication sample of 201 hydrochlorothiazide-treated hypertensives from the PEAR study. No polymorphism was significantly associated with blood pressure response [76].

In the Genetics of Hypertension-Associated Treatment Study (GenHAT), participants of the Antihypertensive and Lipid-Lowering treatment to prevent Heart Attack Trial (ALLHAT) were studied for the association between α-adducin Gly460Trp polymorphism and blood pressure response to chlorthalidone vs other drugs (n = 36,913; 12,696 of African ancestry). Carriers of the Trp allele are reported to have a greater occurrence of salt-sensitive hypertension, and blood pressure response to diuretics was pronounced with the Trp allele, compared to the Gly allele in European ancestry populations [25]. However, GlyGly homozygotes were significantly more frequent in participants of African ancestry than in other participants (82.6% vs 67.4%, *P* <0.01), and there was no significant difference in systolic or diastolic blood pressure response between Trp allele carriers and non-carriers (systolic/diastolic reduction in Trp allele carriers −7.42/–3.23 mm Hg, vs −7.44/–3.57 mm Hg in non-carriers; *P* >0.05) [25].

Also, polymorphisms of the *GRK2* gene (ADRBK1) and *GRK5* Gln41Leu, which are reported to mediate down-regulation of β-adrenergic signaling, were studied in 418 patients (167 of African ancestry) from the PEAR study. The genotypes were not associated with the blood pressure response to hydrochlorothiazide [77].

Finally, using genome wide analysis, good and poor responders to hydrochlorothiazide of African (n = 194) and European ancestry (n = 195) were compared. Variation in one region on chromosome 12q15 emerged to be significantly associated with blood pressure response, but only in subjects of African ancestry [21]. Follow-up analysis favored *YEATS4*, a gene probably encoding a transcription factor, over *LYZ*, encoding lysozyme, as positional candidate genes [21]. The study has now been replicated [83], but the biological mechanism that may underlie the observed associations with blood pressure response to hydrochlorothiazide is hitherto unclear.

### Inhibitors of the RAAS system

#### ACE inhibitors

##### Clinical efficacy

ACE inhibitors are known to induce less blood pressure lowering in patients of African than in European ancestry [7]. In the former, ACE inhibitors do not differ from placebo in achieving diastolic goal blood pressure with monotherapy [3]. The main difference in side effects is the relatively high incidence of angioedema with the use of ACE inhibitors in patients of African ancestry [3].

##### Environmental effects

High salt intake reduces the blood pressure lowering efficacy of ACE inhibition. With a high salt diet (190 mmol sodium/day) and enalapril studied in 391 subjects (96 of African ancestry), systolic blood pressure reduction in mm Hg (SD) was smaller in patients of African, than of European ancestry (respectively, placebo 156.5 (13.1) vs. enalapril 146.2 (16.4); difference −10.3 for African, and placebo 159.2 (13.4) vs enalapril 144.2 (17.5); difference −15.0 for European ancestry groups).

With low salt (88 mmol sodium/day), blood pressure was lower, but the difference persisted (African ancestry, placebo 145.0 (16.1) vs enalapril 137.2 (19.2) difference −7.7; European ancestry placebo 145.1 (17.1) vs enalapril 132.4 (16.2), difference −12.7) [41]. Drug efficacy of ACE inhibitors in patients of African ancestry can thus be modulated by controlling salt intake, or adding thiazide diuretics to the drug regimen [8]. However, even with low salt, the blood pressure lowering effect of ACE inhibitors is greater in patients of European ancestry [41]. This implies that other factors are involved in the difference in drug response.

##### Pharmacokinetics

In the ramipril arm of the AASK study [17], there were no associations between *CYP3A4* A392G, *CYP3A4* T16090C or *CYP3A5* A6986G genotypes and time to reach target mean arterial pressure among men or women randomized to a low or usual mean arterial pressure.

##### Pharmacodynamics

The main mode of action of ACE inhibition is well known, the drugs reduce the activity of angiotensin converting enzyme, and eventually, angiotensin, aldosterone and salt retention. In addition, ACE inhibitors promote NO synthesis in the endothelium [97].

A repressed RAAS system occurs with greater frequency in persons of African ancestry [2,35]. Therefore, any drug further repressing this system could be expected to be less effective in this population group [15,22,38,50]. However, clinical trials have produced mixed results in whether low renin levels adequately predict an attenuated antihypertensive response [22,38,40,50]. As with diuretics, profiling based on age and ancestry was shown to be superior to renin levels in predicting the magnitude of the antihypertensive response to captopril [38].

Regarding the intracellular effect of ACE inhibitors, the drugs were observed to have an ACE independent effect [47], and partly assert their effect through NO [97]. Thus, the lower bioavailability of NO in persons of African ancestry [10,12,36,37,72,79,81], might contribute to the low efficacy of ACE inhibitors. As to the cause of low NO bioavailability, G6PD deficiency [79,80], and low L-Arginine [69,81], associated with enhanced creatine biosynthesis with high creatine kinase [12,72], have been suggested.

G6PD is the first and rate-limiting enzyme of the pentose phosphate pathway, thus serving as the principle source of cellular nicotinamide adenine dinucleotide phosphate-oxidase (NADPH), a cofactor for NO synthase. Vascular endothelial cells constitutively express nitric oxide synthase that forms NO in the presence of oxygen from the semi-essential amino acid L-arginine. NO synthase binds NADPH, flavin adenine dinucleotide, flavin mononucleotide, L-arginine, a heme moiety and tetrahydrobiopterin. Tetrahydrobiopterin synthesis itself is also dependent on available NADPH [80].

In line with this, G6PD deficiency, reported in up to 25% of persons of African ancestry [79], has been shown to reduce NO bioavailability *in vitro*[80]. In addition, the high creatine synthesis associated with the high creatine kinase activity found in persons of African ancestry [11,12,72,73], is thought to hamper the bioavailability of the precursor L-arginine shared with nitric oxide synthase (Figure 2). Thus, high CK has been shown to be associated with low vascular NO bioavailability *in vitro*[72], and L-Arginine was found to be low in persons of African ancestry [69], with supplementation restoring NO bioavailability *in vivo*[81]. However, there are no clinical data yet that associate the response of ACE inhibitors to high CK or low NO.

Pharmacogenomic factors studied include polymorphisms in the *ACE* gene. The *ACE* insertion/deletion genotype *ACE* DD (30% of all participants; 33% of all participants of African ancestry, n = 13,070) had a poorer response to lisinopril treatment than to any of the other three drugs in the GenHAT study. However, the effect was small, a difference of 0.85 mm Hg systolic (SE 0.51) and 0.50 mm Hg diastolic (SE 0.28), with “similar” results reported for the subgroup analysis for patients of African ancestry [28].

In the AASK study, participants randomized to ramipril (n = 347) were genotyped at three polymorphisms on *ACE*, downstream from the *ACE* insertion/deletion polymorphism: G12269A, C17888T and G20037A. Only participants with a homozygous genotype at G12269A and C17888T, and randomized to the usual mean arterial pressure goal (≤107 mm Hg) reached a blood pressure goal significantly faster than those with a heterozygous genotype (adjusted hazard ratio respectively 1.86; 95% CI 1.32 to 3.23, and 1.49; 95% CI 1.01 to 2.13, potentially due to linkage disequilibrium with *ACE* I/D [23].

Finally, in a study including patients of African ancestry (n = 77), functional variants in the promoter region of the angiotensinogen gene (−217G = > A and −20A = > C) were assessed. Patients with the AA genotype of the −217G= > A variant treated with enalapril or lisinopril showed no significant decrease in blood pressure (systolic blood pressure + 0.84 (SD 2.89), *P* = 0.78; diastolic blood pressure −0.47 (SD 1.74), *P* = 0.79); while patients with at least one copy of the −217G allele developed respectively a 7.23 (1.55) and 5.38 (1.12) mm Hg decrease (*P* <0.01). Similarly, in patients with the −20AA genotype no change in blood pressure occurred, whereas in those patients with at least one copy of the −20C allele, systolic blood pressure decreased in response to ACE inhibitor therapy. In line with this, patients with at least one copy of both the −217G and the −20C allele developed substantial decreases in blood pressure (change in mean ambulatory blood pressure, mm Hg: SBP −14.08 +/− 3.72, *P* <0.01; DBP −9.62 +/− 2.74, *P* <0.01) [26].

#### Other drugs affecting the RAAS system

Angiotensin receptor blockers are also less effective in patients of African ancestry as compared to calcium blockers and diuretics [3]. In one study, the mean plasma concentration and elimination half-life of irbesartan were 20 to 25% higher in persons of African than of European ancestry, while the peak plasma concentration was comparable between the two groups [68]. As with ACE inhibitors, ancestry was superior to renin profiling to predict the response to candesartan [22]. Finally, the aldosterone antagonist eplerenone was more effective than losartan in patients of African ancestry, and equally effective as in patients of European ancestry in one trial [84], despite similar or lower plasma aldosterone levels reported in persons of African, compared to European ancestry [29,84]. As stated above, renin levels did not predict the response to spironolactone (100 to 400 mg/d) [65].

#### β-adrenergic blockers

##### Clinical efficacy

The efficacy of systolic blood pressure lowering of β-adrenergic blockade as monotherapy in uncomplicated essential hypertension is not significantly different from placebo in patients of African ancestry, and some trials report significant placebo corrected increase in blood pressure with β-adrenergic blockade in this population group [3,92] The main side effects are metabolic, including higher glucose levels [3].

##### Environmental factors

To our knowledge, there are no environmental factors reported that may help explain the attenuated blood pressure lowering response of patients of African ancestry to β-adrenergic blockade. In the TAIM study, adding sodium restriction (mean 100 mmol/day) to an atenolol regimen (usual/sodium restricted diet: atenolol n = 22/29; placebo n = 26/19) did not enhance the blood pressure lowering effect [70].

##### Pharmacokinetics

Studies on the differences in the pharmacokinetics of β-adrenergic blockers based on ancestry yielded heterogeneous results. Oral clearance of L-propranolol was reported to be similar [56], or higher in persons of African, than in persons of European, ancestry (respectively 28 ml/min/kg, SD 8; vs 21, SD 7; *P* <0.05) [52], with similar, or up to 25% lower peak plasma concentrations [52,56]. In line with this, hepatic metabolism of propranolol via side chain oxidation, 4-hydroxylation or R-propranolol glucuronidation was observed to be higher in persons of African than in those of European ancestry [44]. However, propranolol clearance after intravenous infusion (0.1 mg/kg), was similar in one study [61]. On the other hand, around 30% higher plasma concentrations were found after 100 mg oral metoprolol in an indirect comparison between subjects of African vs European ancestry, respectively 154 ng/ml vs 117 at t = 3 h [59], while others observed no significant differences in plasma peak plasma concentrations or systemic clearance [55]. Also, metabolism of metoprolol via CYP2D6 assessed with an oral dose of 200 mg, given to men of African and European ancestry (10 in each group) was not significantly different [45]. Finally, pharmacokinetic studies of pindolol yielded similar results in both groups [64,67].

##### Pharmacodynamics

The attenuated response of persons of African ancestry to β-adrenergic blockers was extensively studied. As renin lowering contributes to the antihypertensive effect of β-adrenergic blockers, these drugs were expected to be less effective in subjects of African ancestry [8]. Indeed, renin correlated with the blood pressure lowering response to atenolol 50 to 100 mg/d in a study including 67 subjects (33 of African ancestry) [53]. However, renin did not predict the response to propranolol (80 to 640 mg/d) in 215 participants (132 of African ancestry) of a Veterans Administration study [15]. The relative contribution of renin vs African ancestry (β ± SE) was calculated in multivariable regression analysis, to be respectively −4.05 ± 0.84 vs −7.45 ± 1.53; both *P* <0.01 [15]. Finally, in a study of 335 subjects (152 of African ancestry), therapeutic responses to atenolol 25 to 100 mg were consistent with a baseline renin profile, but age-ancestry subgroup profiling was a better predictor of response [38].

β-blockers are thought to lower blood pressure predominantly through a reduction in cardiac contractility and heart rate. While early studies found a reduced sensitivity to isoprenaline in healthy men of African ancestry [49], reports on changes in heart rate after β-blockers in healthy volunteers were conflicting, with either a greater response in persons of African ancestry (to oral propranolol 240 mg/d); [75] an attenuated response (to intravenous propranolol up to 0.15 mg/kg [78], or metoprolol 50 μg/mL); [55] or no significant difference between groups (to intravenous propranolol 0.15 mg/kg) [49]. We retrieved no studies in hypertensives.

Pharmacogenomic studies focussed on the frequency of occurrence of the responsive β1-receptor (*ADRB1*) genotype Arg 389/Ser 49 in persons of African ancestry, which was associated with greater blood pressure lowering responses to β-adrenergic blockade in other population subgroups [14].

In one small study, including 40 subjects (10 of African ancestry) patients homozygous for Arg at codon 389 had a nearly three-fold greater reduction in daytime diastolic blood pressure (−13.3% +/− 8.4% versus −4.5% +/− 8.2%, *P* <0.01) compared with those who carried the variant allele, and Ser49-Arg389/Ser49-Arg389 diplotype demonstrated a decline in blood pressure of 14.7 mm Hg versus 0.5 mm Hg in patients with the Gly49-Arg389/Ser49-Gly389 diplotype, this was independent of ancestry [74].

In addition, Kurnik *et al.* studied sensitivity to β-blockade by the attenuation of exercise-induced tachycardia in 165 subjects (73 of African ancestry), and found that heart rate reduction was greatest in the Arg389/Arg389 group, intermediate in the heterozygotes, and smallest in the Gly389/Gly389 group; this effect was seen in both ancestry groups. Carriers of the responsive Arg389/Ser49 haplotype, had a 27% greater adjusted reduction in heart rate at maximal exercise (mean difference, 3.7 bpm; 95% CI, 1.2 to 6.2; *P* <0.01). However, differences in sensitivity to the β1-blocker atenolol persisted after accounting for different distributions of functional genetic β1-receptor variants, suggesting that additional factors contribute to the differences found between ancestry groups [20].

The AASK study yielded conflicting results as time to reach the target mean arterial pressure of 107 mm Hg with metoprolol (329 participants randomized) was not significantly different for Ser49 or Gly49 variants. In contrast with studies in other population subgroups, the “hazard” ratio of reaching goal blood pressure was lower, 0.68 (95% CI 0.50 to 0.93) in individuals with at least one ‘responsive’ Arg389 allele compared to individuals with Gly389/Gly389 [14].

Finally, a series of pharmacogenomics studies did not further explain why patients of African ancestry respond less to β-adrenergic blockade. The G-protein-coupled receptor kinase 5 (*GRK5*) codes for a serine/threonine kinase that phosphorylates and desensitizes G-protein-coupled receptors. However, in a study of 154 healthy subjects (69 of African ancestry), *GRK5* Gln41Leu polymorphism, present in approximately 40% of the persons of African and 2% of individuals European of ancestry, did not affect the response to atenolol [18]. Furthermore, polymorphisms of the *GRK2* gene (ADRBK1) and *GRK5* Gln41Leu polymorphisms, studied in 418 patients (167 of African ancestry) from the PEAR study did not affect the blood pressure response to atenolol [77]. Finally, the polymorphisms Arg65Leu, Ala142Val, and Ala486Val of the G protein-coupled receptor kinase gene, *GRK4*, were studied in the AASK Study [19]. Only in men randomized to the usual blood pressure goal (mean arterial pressure 102 to 107 mm Hg), the adjusted “hazard” ratio to reach goal blood pressure with metoprolol was 1.54 (95% CI 1.11 to 2.44; *P* <0.01) with Ala142Val. There was no association between *GRK4* polymorphisms and blood pressure response to metoprolol in women. Thus, despite extensive research, there is no clear pharmacogenomic evidence why patients of African ancestry may have a differential response to β-adrenergic blockade.

An important aspect of β-blocker therapy is that inhibition of β2-mediated vasodilation by β-adrenergic blockers may induce peripheral vasoconstriction and blood pressure increase, thus counteracting the antihypertensive effect [3,92]. (Nebivolol, a β-adrenergic blocker that generates intravascular NO is reported to have less vasoconstrictive effect) [16,27]. β2-adrenergic effects were addressed in the following studies. A blunted forearm flow response was reported in subjects of African vs European ancestry after intra-arterial infusion of isoprenaline, a nonselective β-adrenergic agonist (respectively 10.9 (SE 1.7) with African, versus 14.9 (1.5) mL/min/dL with European ancestry; *P <*0.01 [37], with similar results in an independent study [42]. However, lymphocyte β-2-adrenergic receptor density was found similar in subjects of African compared to European ancestry (African, 19.2 +/− 2.2 fmol/mg protein; European, 15.2 +/− 1.4 fmol/mg protein) [54], with a lower affinity of the β2-receptor for propranolol in persons of African ancestry [51].

Studies on differences in intracellular cAMP production as part of the intracellular signaling cascade after receptor stimulation yielded conflicting results. Lower, as well as higher, baseline and isoproterenol stimulated cAMP levels were found in subjects of African compared to European ancestry [54,62], and men of African ancestry with the highest lymphocyte β2-adrenergic agonists mediated cAMP production had the greatest blood pressure increases during antagonist (metoprolol) therapy [48].

The intracellular signaling pathway after β2-adrenergic stimulation and cAMP production eventually leads to inhibition of myosin light chain kinase activity and vasodilation [91]. β-blockers may thus promote peripheral vasoconstriction. Moreover, there is evidence of high vascular smooth muscle creatine kinase in persons of African ancestry. The enzyme CK rapidly provides ATP for enzymes leading to vasoconstriction, including myosin light chain kinase [11,12,72,73]. Hence, high activity of CK may facilitate pressor responses with β-blockers (Figure 2), but as yet there are no clinical data to substantiate this.

### α-1-adrenergic antagonists

There were only minor pharmacokinetic differences between subjects of African (n = 6) and European ancestry (n = 6) in trimazosin pharmacokinetics, with the latter having a larger volume of distribution, and a longer terminal elimination half-life for the metabolite, 1-hydroxy-trimazosin [60]. Furthermore, profiling based on age and ancestry was shown to be superior to renin levels in predicting the magnitude of the antihypertensive response to prazosin [38].

## Discussion

Why do hypertensive patients of African ancestry generally respond better to diuretics and calcium blockers and less well to ACE and β-adrenergic blockade? Many clinicians use the self-defined ancestry of a patient as a clinical guide to select antihypertensive drugs [5], but considerable overlap in response is known to occur between ancestry groups [3,6-8]. Therefore, many health care workers and patients object to using ancestry as a proxy for drug response [7,8], and it is advocated that reduction of blood pressure and related mortality should be achieved through individual treatment options [5,7,8]. However, to reach this end, ethno-cultural and biological differences in drug response behind the surrogate measures of ‘ancestry’ or ‘ethnicity’ need to be identified.

To our knowledge, this is the first systematic review on environmental, pharmacokinetic and pharmacodynamic factors that may contribute to the differential clinical response to different types of drugs observed in patients of African ancestry. In this paper, we also addressed genetic variation thought to affect pharmacokinetic and pharmacodynamic mechanisms, of which phase 1 and phase 2 drug metabolism and receptor function have been most extensively studied.

However, the magnitude of the effects of variation in single candidate genes on antihypertensive drug responses appears to be very modest, accounting for only a small percentage of total variation in response when reported (<5%). Also, we found considerable heterogeneity in the direction of the effect across sex and ancestry groups. Studies of polymorphisms may reflect inheritance of a locus in linkage disequilibrium with the gene variation. Because linkage disequilibrium is affected by the population’s history, true associations due to linkage disequilibrium may yield conflicting results in two separate populations [98]. No unique mutation was by itself predictive of the therapeutic response to these drugs, and even the combined effects of polymorphisms did not account for enough variation in response to be clinically useful.

Differences in pharmacodynamics were most consistent, mainly related to the pathophysiology and clinical characteristic of hypertension in patients of African ancestry. In this regard, new views have developed that expand the classical pathophysiology of patients of African ancestry to have low renin hypertension [2,8]. Low renin in itself does not explain the greater occurrence of hypertension or the enhanced vascular contractility reported in this group [11], and in the presented data, profiling based on age and ancestry was equal or superior to renin in predicting drug responses. Recent data point to a central role for the balance between NO bioavailability and creatine kinase activity [10,12,16,31,72,79-81]. The NO and CK systems share a common precursor in L-Arginine, and display antagonizing effects with mutual inhibition (Figure 2). NO inhibits CK, lowers blood pressure and promotes cardiovascular health [11,12,81,94]. High CK activity is thought to promote salt retention and vascular contractility, with low renin as an epiphenomenon [11,12,72]. Cytoplasmic CK is tightly bound near ATPases, such as Na^+^K^+^ ATPase and myosin ATPase, to rapidly transfer a phosphoryl group from creatine phosphate to adenosine diphosphate (ADP) *in situ*, and generate ATP near these ATPases, thereby facilitating ion transport and muscle contractility [11,12,88-90]. The high creatine synthesis associated with high creatine kinase activity demands L-Arginine, which is thought to lower NO bioavailability [12,72]. In line with this, CK is the main predictor of blood pressure in the general population [11,12,99], and of failure of antihypertensive therapy [100]. Patients of African ancestry are reported to have low NO bioavailability [10], high CK activity [11,12,72,73], and low L-arginine [69], with restored NO bioavailability upon L-Arginine supplementation [81]. However, although it is plausible that inter-individual differences in blood pressure lowering efficacy of drugs could be related to the balance between NO and CK activity, with lower efficacy of drugs that require NO synthesis (such as ACE inhibitors), or promote CK-dependent vasoconstriction (β-adrenergic blockers), and higher efficacy of drugs that counteract CK (diuretics and calcium blockers), there are no further clinical data yet to substantiate this. Hitherto, self-defined ancestry remains the best predictor of responses to antihypertensive drugs, and is shown superior to renin status.

The main strength of this study is that this is the first systemic review, designed to assess potential causes for the different responses of patients of African ancestry to antihypertensive drugs, including all published papers without language restriction, and considering salt intake, recent development in pathophysiology and pharmacogenomics, as well as resulting differences in pharmacokinetics and pharmacodynamics. Our systematic approach reduces over-interpretation of study data, and increases the transparency and reproducibility of the synthesis [13].

Using this rigid methodology, the data on potential predictors of blood pressure response in patients of African ancestry are far less conclusive than in previously published, non-systematic overviews [6,8,98], with self-defined ancestry remaining the best predictor of responses to antihypertensive drugs. Although there is considerable heterogeneity among persons of sub-Saharan African descent, because of observed group differences in risk for hypertension, the field of hypertension continues to treat this group as a distinct biological entity [101]. We included environmental as well as biological factors, but we are aware that in a real world setting, differences in access to care, clinical management and adherence to treatment may have more impact on morbidity and mortality of patients of African ancestry than the differential response to antihypertensive drugs [102]. Still, in our focus on the effect of drug therapy on blood pressure, we address the most practical aspect of treatment. Lowering blood pressure is the most cost-effective way to reduce the morbidity and mortality of hypertension, and choosing highly effective drugs early in the treatment procedure helps achieve early adequate blood pressure lowering and leads to greater adherence [5,8]. We also note that for many patients, this would mean using initial combination therapy [5], but there are insufficient data available to address differences in pharmacokinetic and pharmacodynamics of combination therapy based on ancestry.

## Conclusions

Patients of African ancestry tend to suffer from more severe hypertension, characterized by enhanced vascular contractility and salt retaining capacity, therapy resistance, and higher morbidity and mortality of the condition and its complications. Because of the need for individual treatment options, as well as the increasing objections to the use of ancestry as a surrogate marker for therapeutic responses, we systematically gathered evidence on biomarkers that may predict the response of individual persons of African ancestry to different types of antihypertensive drugs. However, pharmacogenomics yield heterogeneous, insufficient evidence, and the low renin levels found with greater frequency in patients of African ancestry do not, or do not adequately, predict responses to antihypertensive drugs. Finally, there are no convincing clinical data yet of the emerging paradigm that low NO bioavailability and associated high cellular ATP buffer capacity predict the response to specific antihypertensive drugs. Currently, self-identified ethno-geographic ancestry remains the best available predictor of blood pressure lowering responses to antihypertensive drugs.

## Abbreviations

AASK: The African-American study of kidney disease and hypertension; ACE or gene ACE: Angiotensin converting enzyme; ACE-i: ACE inhibitor; ADD1: Alpha-adducin gene; ADP: adenosine diphosphate; ADRB1: β-1-adrenergic receptor gene; AGT: Angiotensinogen gene; AGTR1 or AT1R: Angiotensin II receptor type I gene; AIM: African index medicus; ALLHAT: The antihypertensive and lipid-lowering treatment to prevent heart attack trial; BP: Blood pressure; Ca-blockers or CaB: Calcium channel blocker; cAMP: Adenosine cyclic 3′,5′-(monophosphate); cGMP: Guanosine cyclic 3′,5′-(monophosphate); CK: Creatine kinase; Creatine ~ P: Creatine phosphate; CYP11B2: Aldosterone synthase gene; EMA: European Medicines Agency; FDA: The Food and Drug Administration; GenHAT: The genetics of hypertension-associated treatment study; GNB3 and GNAS1: G-protein subunits genes; HCT: Hydrochlorothiazide; INVEST-GENES: The International Verapamil/trandolapril study (INVEST) genetic substudy; LC: Light chain; LILACS: Literatura Latino-Americana y del Caribe en Ciencias de la Salud; MAP: Mean arterial blood pressure; MLCP: Myosin light chain phosphatase; NADPH: Nicotinamide adenine dinucleotide phosphate-oxidase; NO: Nitric oxide; NOS3: Endothelial nitric oxide synthase gene; PEAR: The pharmacogenomic evaluation of antihypertensive responses; RAAS: Renin**-**ngiotensin**-**aldosterone system; REN: Renin gene; SER: Sarcoendoplasmic reticulum; TAIM: The trial of antihypertensive interventions and management.

## Competing interests

YKS declare to have no competing interests. LMB is an inventor on NL patent WO/2012/138226 (filed).

## Authors’ contributions

Both authors have made substantial contributions to conception and design. LMB performed the search. Both authors contributed to extraction, analysis and interpretation of the data, and to drafting the manuscript. The authors read and approved the final manuscript and have given final approval of the version to be published.

## Pre-publication history

The pre-publication history for this paper can be accessed here:

http://www.biomedcentral.com/1741-7015/11/141/prepub
